# Joint analysis of multiple blood pressure phenotypes in GAW19 data by using a multivariate rare-variant association test

**DOI:** 10.1186/s12919-016-0048-3

**Published:** 2016-10-18

**Authors:** Jianping Sun, Sahir R. Bhatnagar, Karim Oualkacha, Antonio Ciampi, Celia M. T. Greenwood

**Affiliations:** 1 Department of Epidemiology, Biostatistics and Occupational Health, McGill University, Montreal, QC H3A 1A2 Canada; 2 Lady Davis Institute for Medical Research, Jewish General Hospital, Montreal, QC H3T 1E2 Canada; 3 Département de Mathématiques, Université du Québec à Montréal, Montréal, QC H2X 3Y7 Canada; 4 Department of Oncology, McGill University, Montreal, QC H2W 1S6 Canada; 5 Department of Human Genetics, McGill University, Montreal, QC H3A 1B1 Canada

## Abstract

**Introduction:**

Large-scale sequencing studies often measure many related phenotypes in addition to the genetic variants. Joint analysis of multiple phenotypes in genetic association studies may increase power to detect disease-associated loci.

**Methods:**

We apply a recently developed multivariate rare-variant association test to the Genetic Analysis Workshop 19 data in order to test associations between genetic variants and multiple blood pressure phenotypes simultaneously. We also compare this multivariate test with a widely used univariate test that analyzes phenotypes separately.

**Results:**

The multivariate test identified 2 genetic variants that have been previously reported as associated with hypertension or coronary artery disease. In addition, our region-based analyses also show that the multivariate test tends to give smaller *p* values than the univariate test.

**Conclusions:**

Hence, the multivariate test has potential to improve test power, especially when multiple phenotypes are correlated.

## Background

In many clinical or epidemiological studies, multiple measures of related traits, that is, a multivariate phenotype, are collected. For example, in the Genetic Analysis Workshop 19 (GAW19) data [1], both systolic and diastolic blood pressures are available for each subject. It is possible that these related traits share some common genetic architecture either through pleiotropy—one genetic variant influencing multiple traits [2, 3]—or by contributions of different variants in the same gene [4]. Under such situations, multivariate methods may help in genetic association studies, as they may add ability to investigate the genetic architecture, and increase power to detect disease-associated loci [5].

Various methods for assessing associations between a single genetic variant and multiple traits jointly have been developed (summarized in Yang and Wang [6]). However, individual variant tests can have limited power to detect association with rare variants (with minor allele frequency [MAF] less than 5 %). Consequently, region-based tests have become a standard alternative approach to summarize the genetic variability of a set of rare variants in a defined region [7–9].

Recently, Sun et al. [10] proposed a novel region-based multivariate test, MURAT (Multivariate Rare-variant Association Test), for identifying rare-variant associations when multiple correlated continuous phenotypes are observed. By assuming the variant effects to be randomly distributed yet correlated, and allowing arbitrary correlations among phenotypes, MURAT is more general than the other comparable multivariate methods. In addition, MURAT shows potential to improve test power especially when there are pleiotropic effects or highly correlated phenotypes. In this study, we apply MURAT to the GAW19 sequencing data for unrelated samples to identify variants that are associated with blood pressure. In addition, we also compare MURAT with the sequence kernel association test (SKAT) [11], a widely used univariate test for rare variant association.

## Methods

### Joint analysis of multiple phenotypes

Here we briefly summarize the MURAT method, which is described in more detail in Sun et al. [10]. Suppose for each subject *i*, *K* correlated phenotypes, ${\boldsymbol{Y}}_i={\left({Y}_{i1},\dots, {Y}_{iK}\right)}^T$, are observed, and we are interested in detecting variants that are associated with these phenotypes. For most region-based tests, a single phenotype, ${Y}_{ik}$, is linked with the genotype values of a group of *v* SNPs, ${\boldsymbol{G}}_i={\left({G}_{i1},\dots, {G}_{iv}\right)}^T$, and *m* covariates, ${\boldsymbol{X}}_i={\left({X}_{i1},\dots, {X}_{im}\right)}^T$, via a linear model ${Y}_{ik}={\alpha}_{0k}+{\boldsymbol{\alpha}}_k^T{\boldsymbol{X}}_i+{\boldsymbol{\beta}}_k^T{\boldsymbol{G}}_i+{\varepsilon}_{ik}$ for $k=1,\dots, K$, where ${\alpha}_{0k}$ is a scalar for intercept, ${\boldsymbol{\alpha}}_k={\left({\alpha}_{k1},\dots, {\alpha}_{km}\right)}^T$ and ${\boldsymbol{\beta}}_k={\left({\beta}_{k1},\dots, {\beta}_{kv}\right)}^T$ are corresponding coefficient vectors, and ${\varepsilon}_{ik}$ is a random error, assumed to follow a standard normal distribution.

For MURAT, however, *K* phenotypes are associated with genotype and covariates jointly by using a multivariate linear model, ${\boldsymbol{Y}}_i={\boldsymbol{\alpha}}_0+\left({I}_K\otimes {\boldsymbol{X}}_i^T\right)\boldsymbol{\alpha} +\left({I}_K\otimes {\boldsymbol{G}}_i^T\right)\boldsymbol{\beta} +{\boldsymbol{\varepsilon}}_i,$ where ${\boldsymbol{\alpha}}_0={\left({\alpha}_{01},\dots, {\alpha}_{0K}\right)}^T$, $\boldsymbol{\alpha} ={\left({\alpha}_{11},\dots, {\alpha}_{1m},\dots, {\alpha}_{K1},\dots, {\alpha}_{Km}\right)}^T={\left({\boldsymbol{\alpha}}_1^T,\dots, {\boldsymbol{\alpha}}_K^T\right)}^T$, $\boldsymbol{\beta} ={\left({\beta}_{11},\dots, {\beta}_{1v},\dots, {\beta}_{K1},\dots, {\beta}_{Kv}\right)}^T={\left({\boldsymbol{\beta}}_1^T,\dots, {\boldsymbol{\beta}}_K^T\right)}^T$, ${\boldsymbol{\varepsilon}}_i={\left({\varepsilon}_{i1},\dots, {\varepsilon}_{iK}\right)}^T$, ${I}_K$ is a $K\times K$ identity matrix, and ⊗ represents Kronecker product. The variant effect, ***β***, in the above multivariate model is assumed to be normally distributed, $\boldsymbol{\beta} \sim {N}_{Kv}\left(0,{\varSigma}_{\beta}\right)$. In addition, in order to account for the correlations among ${Y}_{ik}$’s, the unknown matrix ${\varSigma}_{\beta }$ is assumed to have a specific correlation structure, such that there is correlation between ${\boldsymbol{\beta}}_k$ and ${\boldsymbol{\beta}}_{k^{\prime }}$ for $k\ne {k}^{\prime }$. That is, there is a common correlation for the effects of the same variant on different phenotypes, $Corr\left({\beta}_{kj},{\beta}_{k^{\prime }j\;}\right)=\rho$ for variant *j*, but the effects of different variants are uncorrelated, so that $Corr\left({\beta}_{kj},\;{\beta}_{k^{\prime }{j}^{\prime }}\right)=0$ for variant *j* and variant *j*’. A score type statistic is derived for MURAT to test whether ***β*** equals zero. With a data-adaptive procedure to manage unknown correlations among variant effects, ${\boldsymbol{\beta}}_k$, MURAT calculates the *p* values analytically, and hence is fast enough to be used for a genome-wide analysis.

### Phenotypic and sequencing data

In this study, we focused on the 1943 unrelated Mexican American samples provided by GAW19, and considered 2 phenotypes, systolic blood pressure (SBP) and diastolic blood pressure (DBP). Age and sex were used as covariates. After removing subjects who have one or both missing phenotypes, a total of 1851 individuals were considered in the analysis. We applied a log transformation to SBP and DBP so as to eliminate skewness; the correlation between log SBP and log DBP was 0.542.

### Single-variant tests

Although MURAT and SKAT were designed for region-based rare-variant association studies, they can also be used for single-variant tests. Hence, we selected all exomic variants of the odd-numbered chromosomes provided by GAW19. However, because a lot of these variants are very rare, possibly observed only once or twice, we restricted analysis to only the variants that had 4 or more carriers. As a consequence, a total of 152,337 single-nucleotide polymorphisms (SNPs) were included. Missing genotype values were imputed by the corresponding variant MAFs so that the MAFs didn’t change after imputation.

### Region-based tests

To explore the performance of MURAT as a region-based test, we also applied it to various predefined variant sets. We first used the *hg19* reference as the annotation file (see https://www.cog-genomics.org/static/bin/plink/glist-hg19) to obtain gene start and gene end positions to define gene-based regions, and then implemented MURAT and SKAT for each gene. In total, 10,886 genes that contain variants in the unrelated GAW19 genotype data were included in the analysis. We also analyzed the GAW19 data using a series of non-overlapping windows of 30 kb, spanning all provided SNPs, which yielded 13,094 windows, and applied MURAT and SKAT to these mutually exclusive regions. Moreover, we performed a focused comparison of MURAT with SKAT for 15 genes that have been reported as associated with hypertension at *p* < 1.0 $\times$10^−5^ according to the National Institutes of Health (NIH) Genome-Wide Association Studies (GWAS) catalog [12].

## Results

For single-variant tests, to adjust for multiple testing, a Bonferroni-corrected threshold, *p*<0.05/152,337=3.28 $\times$ 10^−7^ would be needed. There were 4 SNPs where MURAT’s *p* value met this level of significance. However, this threshold is likely to be conservative as it assumes independence of all tests. Therefore, Table 1 lists all SNPs with *p* values less than 10^−6^ obtained by either SKAT or MURAT. The MURAT *p* values are smaller than either of the two SKAT *p* values at 7 of the 8 SNPs in Table 1.

**Table 1:** Results of single-variant association tests for all SNPs where either MURAT or SKAT (of either phenotype) showed evidence of association at *p*<10^−6^

				*p* Values		
				SKAT		MURAT
chr	rsID	Nearest gene	MAF	SBP	DBP	BOTH
1	rs4926600	*CYP4A22*	0.0825	3.05e-03	1.39e-01	1.10e-07
1	var_1_91818773		0.0011	3.83e-01	8.23e-04	8.51e-07
3	rs79314450	*HRH1*	0.0014	4.95e-02	4.21e-03	1.75e-08
7	rs116690173	*INMT*	0.0016	3.12e-01	2.47e-03	6.23e-07
7	rs79584800	*FAM188B*	0.0014	2.91e-01	8.14e-04	2.61e-07
17	var_17_61987931		0.0011	9.34e-07	6.08e-04	6.26e-06
19	rs138635091	*FBN3*	0.0016	1.94e-01	6.33e-04	3.11e-08
19	rs115045946	*APOC4*	0.0022	1.09e-01	1.51e-02	5.10e-07

*MAF* minor allele frequency

**Fig. 1:**
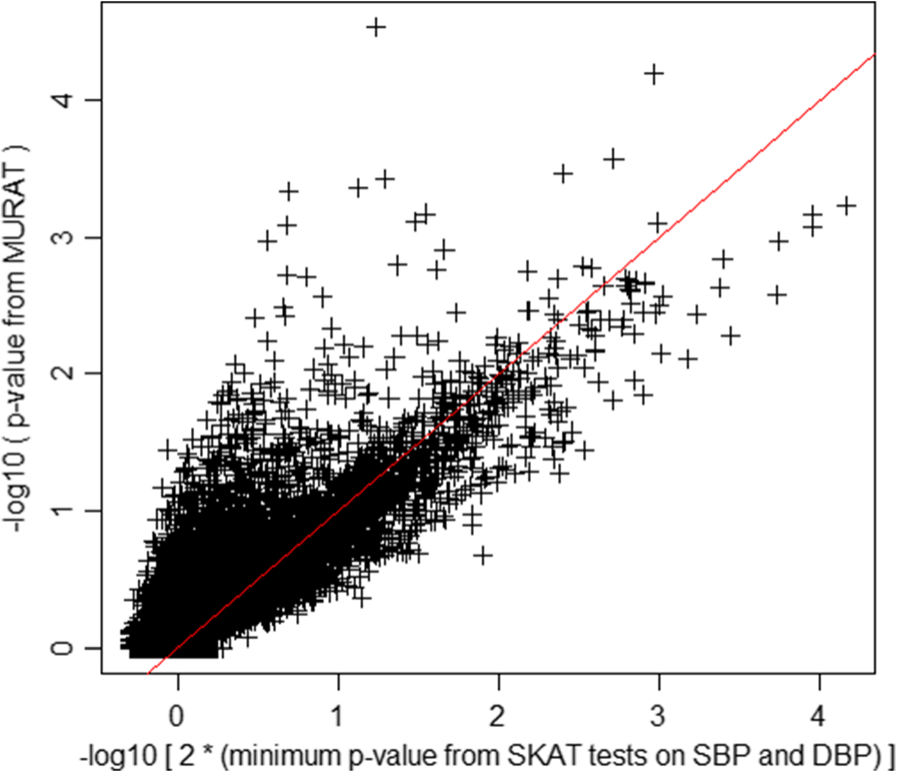
Comparison of −log10 *p* values between MURAT and univariate tests for region-based analysis of each of 10,866 genes. For the single-phenotype tests, a Bonferroni correction was applied to the minimum of the 2 *p* values

**Fig. 2:**
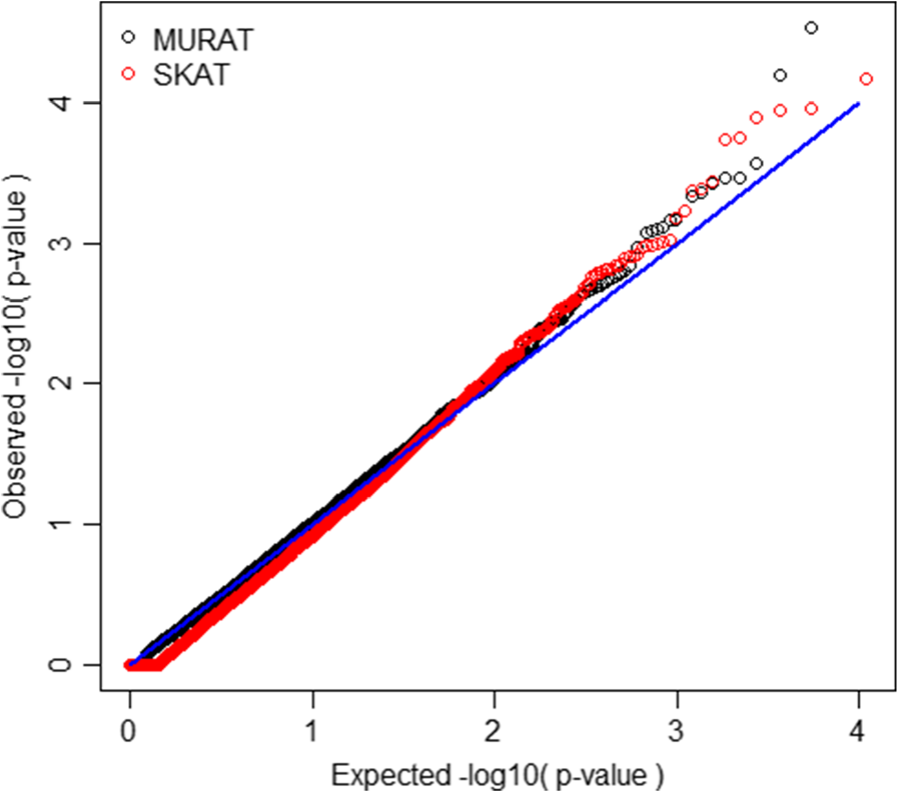
Quantile–quantile (Q-Q) plot for *p* values obtained by using MURAT and adjusted *p* values obtained by using SKAT to test 10,866 genes. The adjusted *p* values are defined as twice the minimum of the SBP and DBP-based *p* values obtained via SKAT

The region-based *p* values obtained from applying MURAT and SKAT to 10,866 genes are plotted in Fig. 1; for SKAT, twice the minimum of the SBP and DBP-based *p* values are shown. Figure 1 clearly shows that MURAT *p* values tend to be smaller than the adjusted SKAT *p* values. In addition, in Fig. 2 we also show the corresponding quantile–quantile (Q-Q) plots for MURAT and SKAT, respectively. Because the Q-Q plots show that *p* values obtained from both MURAT and SKAT follow the expected distribution under the null, we can draw the conclusion from Fig. 1 that MURAT has potential to improve test power over single-phenotype tests. A Bonferroni threshold here would require *p*<4.6 $\times$ 10^−6^ but no genes met this threshold. Using a more liberal threshold of *p*<10^−4^, Table 2 displays all genes where evidence of association was identified with either SKAT or MURAT. In our analysis of 13,094 non-overlapping windows of 30 kb, no regions showed significance at a Bonferroni-corrected level of 3.8 $\times$ 10^−6^, or a more liberal threshold of *p*<10^−5^.

**Table 2:** Results of region-based analysis of all genes where either MURAT or SKAT (of either phenotype) showed evidence of association at *p*<10^−4^

			*p* Values		
			SKAT		MURAT
chr	Gene	# of SNPs	SBP	DBP	BOTH
1	*HIST3H2BB*	4	5.42e-04	1.07e-02	6.43e-05
3	*MIR425*	1	2.95e-02	1.70e-01	2.92e-05
3	*TPRG1-AS2*	3	2.62e-01	5.55e-05	6.79e-04
9	*ZBTB43*	21	4.85e-02	9.20e-05	2.61e-03
15	*ZNF280D*	63	1.03e-02	8.94e-05	1.07e-03
17	*SAT2*	17	4.31e-03	5.52e-05	8.29e-04
17	*SHBG*	58	5.54e-03	3.40e-05	5.85e-04

**Fig. 3:**
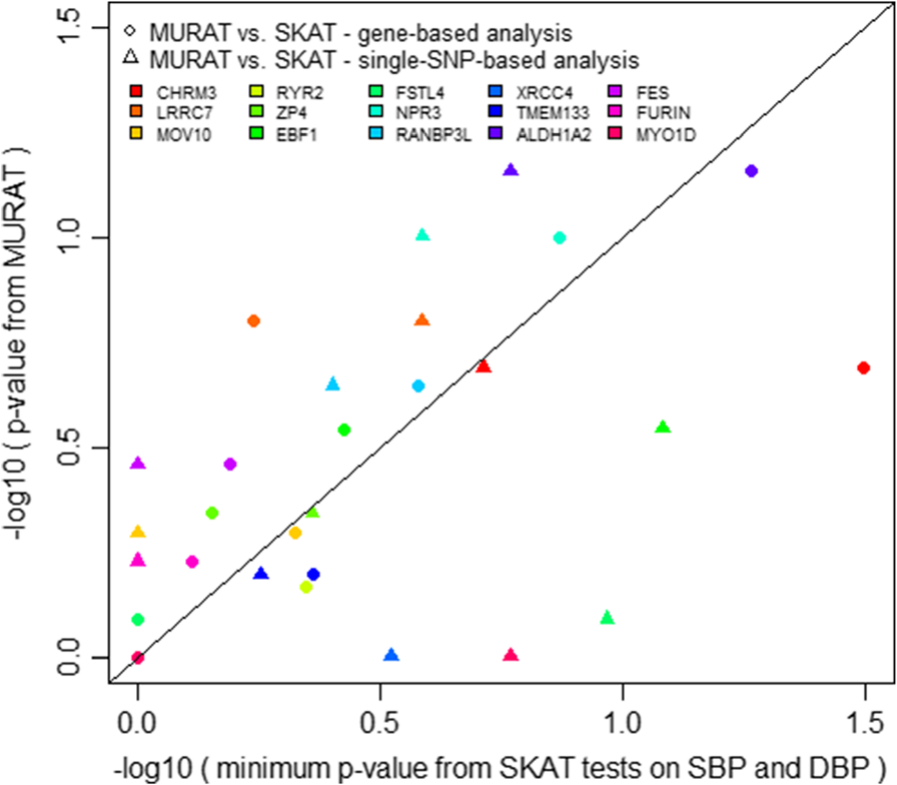
Comparison of *p* values testing for association between the gene-based MURAT test, and single-phenotype tests with either gene-based or single-SNP–based analyses, for 15 known hypertension-associated genes selected from the NIH-GWAS catalog, and occurring on odd-numbered chromosomes. For the single phenotype tests, Bonferroni corrections were applied to adjust the *p* values for testing 2 traits and, for single-variant test results, also for testing all SNPs in a gene

Figure 3 compares the gene-based multivariate MURAT with single-phenotype analyses for 15 genes where association with hypertension has been well demonstrated in the NIH-GWAS catalog. Although none of these genes show strong evidence of statistical significance in these data, some stronger single-SNP–single-phenotype associations are decreased when using MURAT. However, most of the points in Fig. 3 fall above the diagonal line, suggesting a possible power benefit after adjustment for multiple testing. Hence, it may be a beneficial strategy to incorporate a multivariate test into the analysis plan when designing or planning a study.

## Discussion

In this study, we investigated the performance of a joint analysis of multiple phenotypes for genetic association studies by applying MURAT, a novel region-based multivariate test for rare variants, to the GAW19 unrelated samples. The results show that when multiple phenotypes are correlated, the multivariate test can be more powerful than the univariate test.

For the most significant SNPs listed in Table 1, MURAT often estimated significance levels substantially smaller than the single-phenotype analyses; hence it may be more powerful than SKAT. For the one notable exception to this pattern in Table 1 (var_17_61987931 on chromosome 17), there was strong association only with SBP. If a variant is only associated with one of the phenotypes, a joint analysis with the other traits will add noise to the multivariate test.

Some of the SNPs in Table 1 are in or near genes that are good candidates for hypertension. In particular, SNP rs4926600 on chromosome 1 is within gene *CYP4A22*, which was previously reported to be associated with essential hypertension [13, 14]. Similarly, SNP rs115045946 on chromosome 19 is near to gene *APOC4*, which is associated with coronary artery disease [15]. Comparing gene-based tests (see Table 2) with single SNP tests (see Table 1), we found that the region based tests in this study did not seem to demonstrate improved power over the single variant tests. This has also been found by others [16, 17], and is likely because the region based tests are sensitive to the proportion of causal variants in a region and lose power when many neutral variants are included.

Hence, power for region-based tests should be best when there are many causal variants. To investigate power of this multivariate test in a situation where associations are likely to be real, we focused on 15 known hypertension associated genes (occurring on odd-numbered chromosomes). Figure 2 suggests that there may be benefit to multivariate region-based tests, if adjustments for multiple testing are applied, and if there is some association with all of the phenotypes.

We have shown [10] that compared with the univariate tests, the MURAT approach is more powerful, especially when there exist pleiotropic effects or highly correlated traits, but it is also subject to possible power loss when many neutral variants exist, or when variants are only associated with a subset of all traits. Hence, for joint analysis of multiple phenotypes, valuable future work should include developing reliable methods to select the best genetic regions for analysis, and the possibility of combining the univariate and the multivariate test together in an optimal manner, in order to ensure the best power under various genetic architectures.

## Conclusions

The new multivariate rare variant test MURAT demonstrated interesting results in joint analysis of systolic and diastolic blood pressure phenotypes in GAW19 unrelated individuals, and identified some loci that are plausible candidates for association with hypertension.
